# PET Radiomics in NSCLC: state of the art and a proposal for harmonization of methodology

**DOI:** 10.1038/s41598-017-00426-y

**Published:** 2017-03-23

**Authors:** M. Sollini, L. Cozzi, L. Antunovic, A. Chiti, M. Kirienko

**Affiliations:** 1 Department of Biomedical Sciences, Humanitas University, via Manzoni, 113-20089 Rozzano, (Milan) Italy; 2 Radiotherapy and Radiosurgery Unit, Humanitas Clinical and Research Center, via Manzoni, 56-20089 Rozzano, (Milan) Italy; 3 Nuclear Medicine Unit, Humanitas Clinical and Research Center, via Manzoni, 56-20089 Rozzano, (Milan) Italy

## Abstract

Imaging with positron emission tomography (PET)/computed tomography (CT) is crucial in the management of cancer because of its value in tumor staging, response assessment, restaging, prognosis and treatment responsiveness prediction. In the last years, interest has grown in texture analysis which provides an “*in-vivo*” lesion characterization, and predictive information in several malignances including NSCLC; however several drawbacks and limitations affect these studies, especially because of lack of standardization in features calculation, definitions and methodology reporting. The present paper provides a comprehensive review of literature describing the state-of-the-art of FDG-PET/CT texture analysis in NSCLC, suggesting a proposal for harmonization of methodology.

## Introduction

Positron emission tomography (PET)/computed tomography (CT) using the radiopharmaceutical ^18^F-fluoro-deoxy-glucose (FDG) has a paramount role in the management of cancer patients owing to its value in tumor staging, response assessment, and restaging as well as in prognosis and prediction of treatment response. The standardized uptake value (SUV) obtained from FDG-PET scans is the most widely used parameter for lesion characterization and it has been shown to have a prognostic value^1^. More recently, volumetric parameters, including metabolic tumor volume (MTV) and total lesion glycolysis (TLG), have also been proposed for assessment of prognosis^2–5^. Moreover, in recent years there has been emerging evidence that the heterogeneity of density values on CT and of FDG uptake within the primary tumor can permit *in vivo* lesion characterization and provide predictive information in malignances, including non-small cell lung cancer (NSCLC)^6–10^.

The term “heterogeneity” conveys different meanings depending on the imaging modality: in FDG-PET it refers to the variability in the distribution of radiopharmaceutical uptake, while in CT it refers to the variability in tissue density. Lesion “heterogeneity” can be described by a multitude of mathematical methods that, taken together, constitute the “texture analysis” which provides numerous quantitative and semiquantitative indices, termed “features”^11–13^. This approach as a whole is named “radiomics”.

Textural features seem to perform better than the conventional uptake parameters used for image interpretation in clinical routine, such as SUV measurements (e.g., SUV_max_, SUV_mean_), which are subject to several limitations^14^. Numerous studies have explored the additional information that can be extracted by texture analysis, with the aim of characterizing tumor lesions. However, these investigations have had multiple drawbacks and limitations, especially with respect to lack of standardization in feature calculation, definitions, and reporting methodology^15^.

Texture analysis has the potential to impact on patient management if its ability to characterize lesions *in vivo* and to provide predictive information is demonstrated in prospective studies. As lung cancer is the fourth most frequently diagnosed malignancy in Europe and the the leading cause of cancer mortality (http://eco.iarc.fr/eucan), texture analysis in such patients, when validated, will strongly impact on patient management and healthcare systems. The present article provides a comprehensive review of the literature describing the state-of-the-art in FDG-PET/CT texture analysis methods in lung cancer. It also reports on the ability of textural features to identify tumor phenotype and to provide additional predictive and prognostic information in patients with NSCLC. Moreover, a comprehensive review of calculation methods, feature names, and definitions is performed, and a scheme for harmonization of methodology and reporting of results is proposed.

## Methods

From the PubMed/MEDLINE database a search algorithm based on a combination of the following terms was used: (a) “texture” or “textural” or “radiomics” or “heterogeneity” or “heterogeneous” or “features” or “histogram” and (b) “lung cancer” or “NSCLC” and (c) “PET” or “PET/CT”. No start date limit was used and the search extended to 23 April 2016. To expand our search, references of the retrieved articles were also screened. Two authors independently searched articles and performed an initial screening of identified titles and abstracts. All studies or subsets in studies investigating the role of PET or PET/CT radiomics in patients with suspected/definite NSCLC were considered eligible. The exclusion criteria were: (a) articles not within the field of interest; (b) review articles, editorials or letters, comments, and conference proceedings; (c) articles not in the English language; (d) case reports or small case series (<10 patients); and (e) *in vitro* or animal studies.

**Figure 1:**
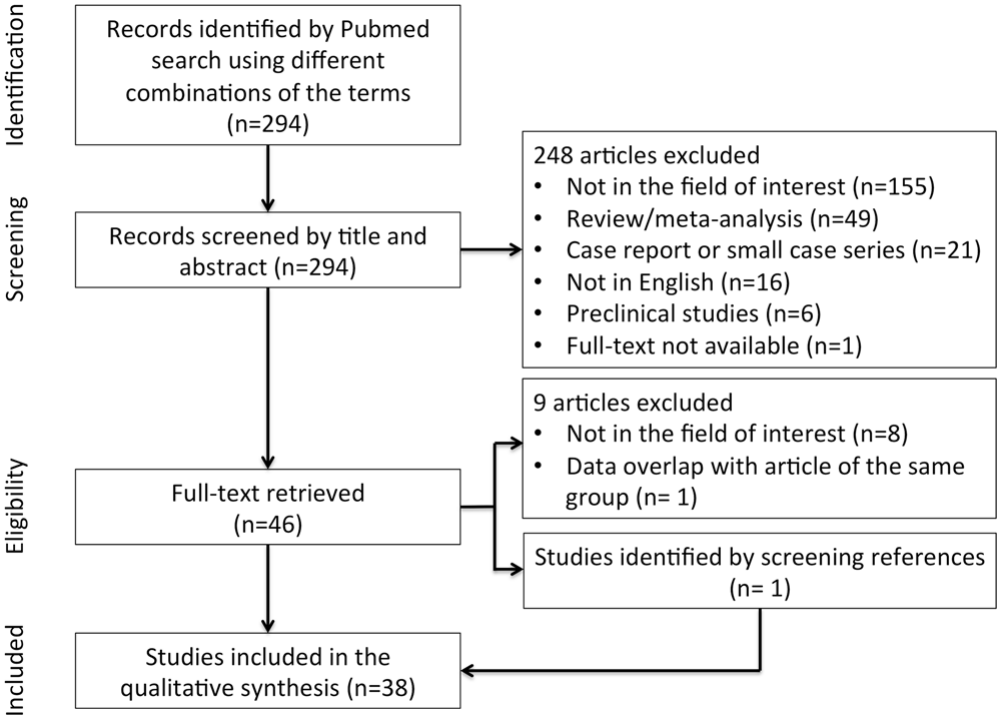
Schematic representation of the process of selection of literature data included in the review.

**Figure 2:**
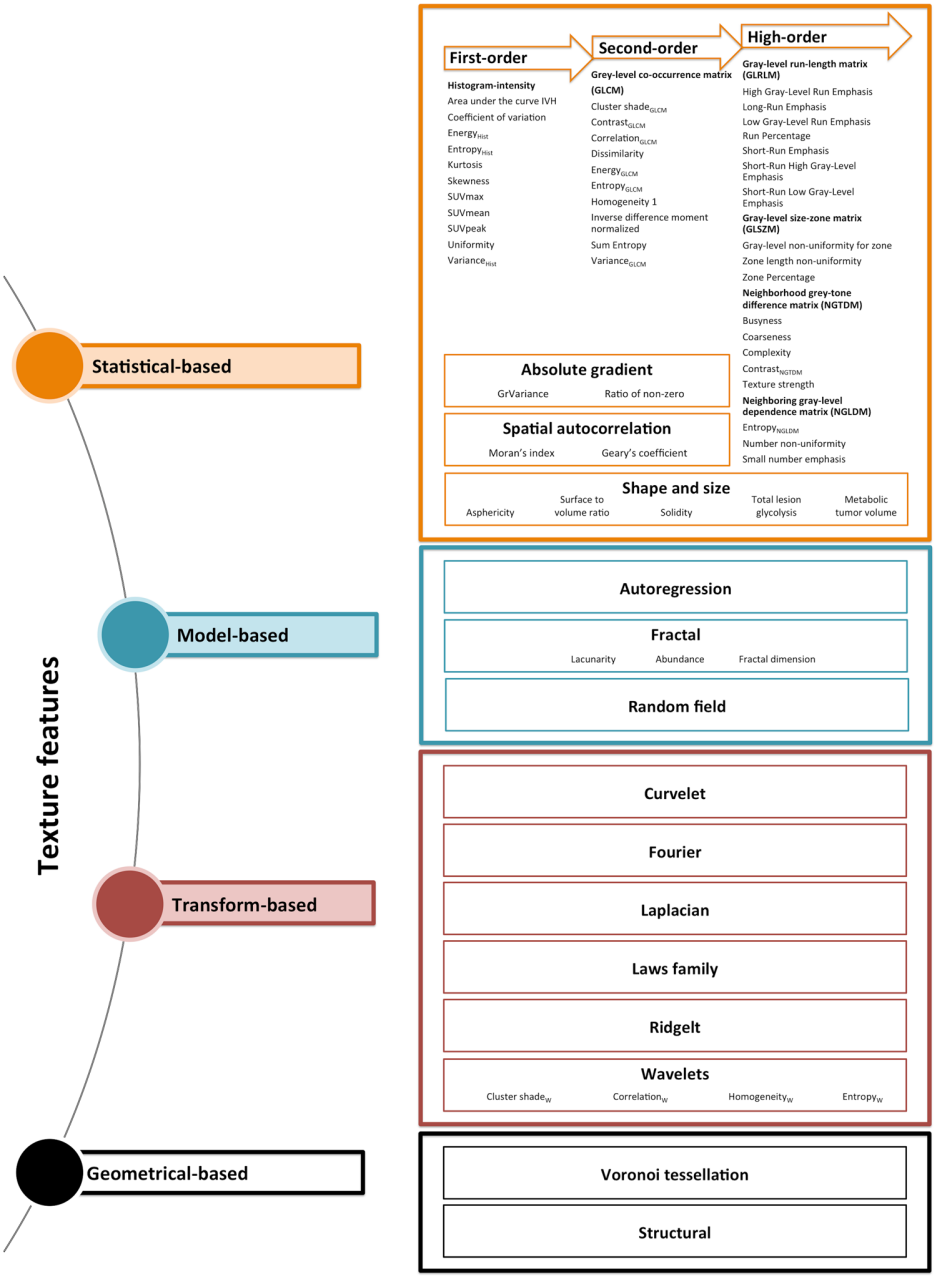
Methodological approaches in image texture analysis (the most frequently evaluated PET features in lung cancer patients are reported as examples).

Among the 294 studies identified by reviewing titles and abstracts, 247 were excluded by applying the criteria mentioned above. One paper not retrievable in the full-text version was excluded. Forty-six articles, retrieved in full-text version, were assessed for eligibility. Nine articles were excluded after reading the full text version. One further article was identified after screening of the references. Overall, 38 articles were selected and used for the qualitative synthesis (Fig. 1). Considering the aim of this review (i.e. texture analysis in NSCLC) which takes into account a variety of heterogeneous papers in terms of aim(s), methods, and results; a systematic review according to the PRISMA algorithm^16^ was considered not feasible. Selected papers were grouped into two different sets based on their aims: technical/methodological and clinical studies. The technical/methodological group included 16 papers (568 patients) that tested specific algorithms, different approaches for segmentation and tumor volume delineation, modalities of image acquisition, attenuation correction, or reconstruction. The clinical set comprised 22 papers (2306 patients), and we separately reviewed the results in respect of the diagnostic and the prognostic or predictive role of the textural features in NSCLC. Although prognosis and treatment prediction should be reported separately, in order to avoid an overlap of contents we treated these topics in the same section since many of the analyzed studies have evaluated them simultaneously. In approaching this review, the main difficulties were related to the differences in textural feature nomenclature and to the comparison of results regarding features obtained using different approaches. Therefore, we started with a reclassification of each feature (Fig. 2). For each feature we specified the order (i.e., first, second, or superior), the matrix (e.g., histogram, gray-level co-occurrence), the definition, and/or the formula, when available, as reported in the Supplementary material. Hereafter the features that are denominated in the identical way but can be derived using different approaches (e.g., histogram, gray-level co-occurrence matrix) are reported by adding the matrix from which they have been derived in subscript.

## Results

### Nomenclature and methods in texture analysis

Texture analysis refers to a variety of mathematical methods that may be applied to describe the relationships between the intensity of pixels or voxels and their position within an image. An advantage of measuring textural parameters is that it is a post-processing technique that can be applied to data acquired during routine clinical imaging protocols, thereby maximizing the information that can be derived from standard clinical images^14^. Distinct approaches (statistics based^17, 18^, model-based^19–21^, transform-based^22–24^, and structural^25^) may be used to analyze functional imaging information, resulting in numerous radiomics features, such as descriptors of the image intensity histogram, “shape and size” features, descriptors of the relationships between image voxels (e.g., GLCM-, and NGTDM-derived features), textures extracted from wavelet and Laplacian of Gaussian filtered images, and fractal features^26^, as shown in Fig. 2.

The first approach consists in the summarizing of 3D functional imaging data into a single curve – histogram – representing the voxel intensity values contained within the volume of interest (VOI), allowing for a simplified interpretation. Intensity-volume histograms (IVH) or cumulative SUV-volume histograms (CSH) have been proposed by El Naqa *et al*.^27^ as a novel way to characterize heterogeneity in tumor tracer uptake. These histograms are similar to dose-volume histograms frequently used in radiotherapy^28^. A set of metrics are derived from IVH representations that reflect the voxel value frequency distribution^27^: I_x_ (minimum intensity to x% highest intensity volume); V_x_ (percentage volume having at least x% intensity value); and descriptive statistics (mean, minimum, maximum, standard deviation, etc.)^29^. In CSH the percent volume of a tumor (derived from CT or from PET-based (semi-)automatic tumor delineation methods^30^) with an SUV above a certain threshold is plotted against that threshold value, which is varied from 0 to 100% of SUV_max_. The area under the CSH (AUC-CSH) may be a quantitative index of tracer uptake heterogeneity and/or heterogeneous tumor response^31^. Any method to characterize heterogeneity, however, will treat both partial volume effect and noise as heterogeneity. Therefore partial volume correction (PVC) and image denoising should be applied prior to calculating AUC-CSH^32^. Despite this consideration, PVC has not been routinely applied. To overcome this potential limitation on the quantitative measurements, most studies have considered relatively large lesions (generally volumes >3–5 cm^3^), assuming that PET cannot characterize heterogeneity in smaller volumes because of its limited spatial resolution.

IVH and other first-order approaches are limited by their spatial insensitivity. To overcome this drawback, textural features and “shape and size” attributes may be extracted that contain embedded spatial and topological information. In fact, second-order and high-order statistics (i.e., based on gray-level matrix, nearest neighbor spatial dependence matrices, etc.) provide information from the spatial relationship of image voxels^33^. The gray-level co-occurrence matrix features may be used to represent texture information because of its relatively simple and intuitive structure. Surface plots of the co-occurrence matrix give a pictorial representation of the spatial-intensity distribution, which is typically masked by first-order histogram analyses. Several other matrices are also used, including the neighborhood gray-tone difference matrix, which provides information regarding how each voxel value differs from the neighboring voxel values; the gray-level run length matrix, which stores the number of voxels with identical values in each direction; and the gray-level size zone matrix, which stores the size of the 3D region that includes a given voxel value^27, 29, 33–37^. Therefore, a multitude of metrics may be derived from the gray-level matrices to characterize the structure of interest^27^. These metrics are independent of tumor position, orientation, size, and brightness and take into account the local intensity-spatial distribution^33, 38, 39^. Hence, the combination of these features can provide an intensity-spatially dependent map of the tumor metabolic uptake that can potentially be used as a signature to characterize the tumor phenotype and response to treatment.

Texture feature extraction requires the voxel intensity values within the VOI to be discretized. This discretization step not only reduces image noise, but also normalizes intensities across all patients, allowing for a direct comparison of all calculated textural features between patients. Shape and size features are calculated, describing the 3D shape and size of the lesions^40^. However, it should be considered that volumetric indexes (e.g., sphericity) may also be extracted from the IVH^40–42^, allowing for a simplified interpretation^27^.

The majority of texture features that have been used in PET medical imaging to date fall into one of the following three categories: (a) first-order features derived from statistical moments of the image intensity histogram, (b) second-order features derived from the GLCM, and (c) higher order features derived from analysis of the NGTDM, NGLDM, or GLSZM^43^. Despite the difficulties in generalization due to the variability in textural PET features among studies, related to the methodology used, we can summarize that IVH features tend to depend on the tumor delineation method^44–46^ and that features derived from GLSZM have been reported to be the most susceptible to variability^40, 46, 47^, while the GLCM-derived features tend to be the most robust. In particular, “entropy_Hist_” and “entropy_GLCM_” have been reported to be features less dependent on the tumor segmentation method^46^, reconstruction settings, iteration numbers, and voxel size^47^, and type of acquisition (3D *versus* 4D)^42, 48^.

### Texture analysis and technical/methodological investigations

**Figure 3:**
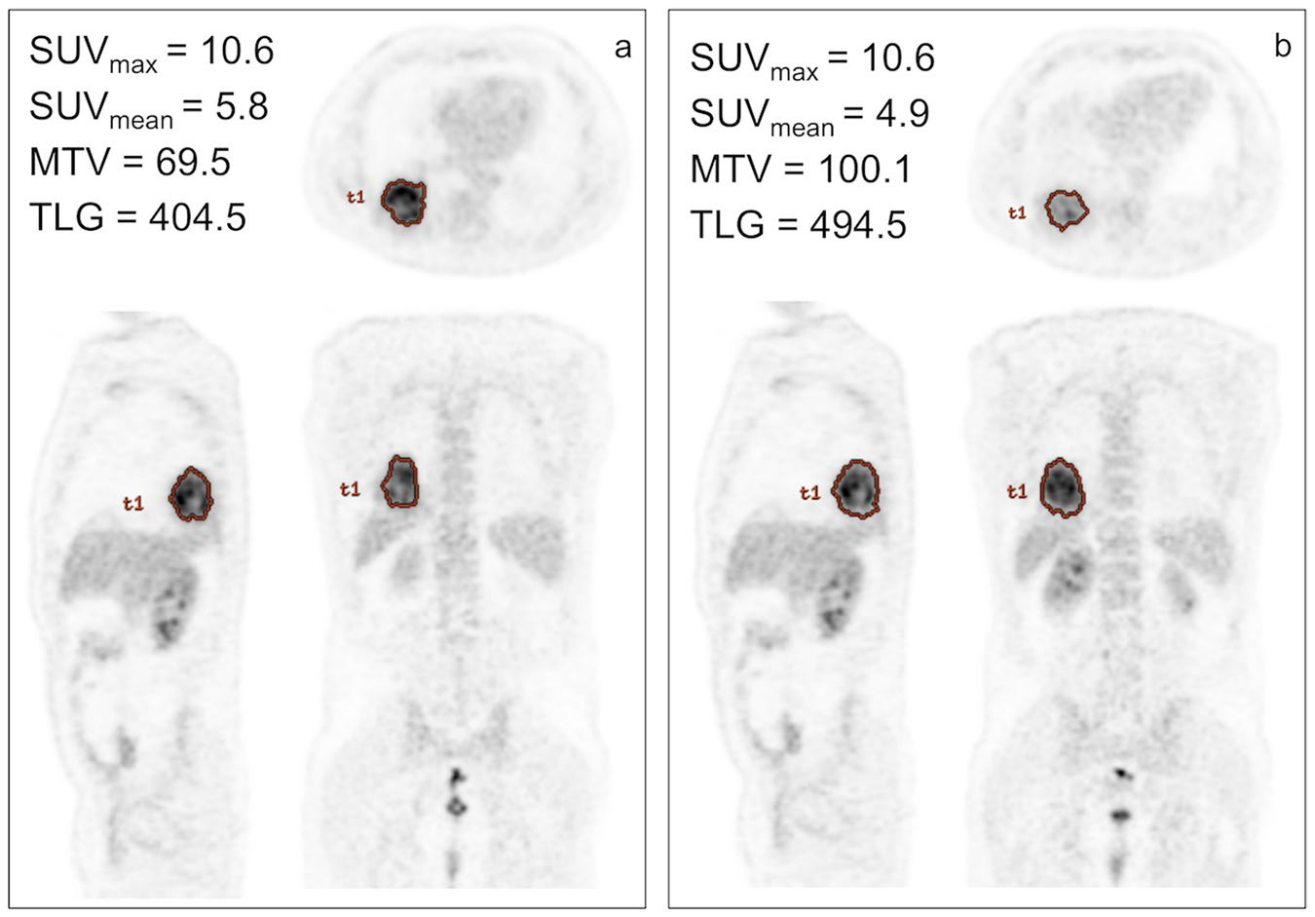
Example of tumor contouring using in (**a**) a threshold method at 50% of SUV_max_ and (**b**) a method based on an absolute SUV cut-off of 2.5. The ROI identified by using the absolute SUV cut-off of 2.5 is greater than that identified by the threshold method, as shown by axial (top), sagittal (right), and coronal (left) images (same slices).

The accuracy and precision of texture analysis derived from PET images depends significantly on scanning protocols. Factors such as image acquisition, reconstruction, and inherent image quality parameters (noise, motion artifacts, and slice thickness) may be important. It is to be expected that all texture analysis methods are influenced to some extent by these factors and the sensitivity of various textural features may be based on different image models. Further aspects that require careful assessment are the methods used for region of interest (ROI) definition on PET images and the intraobserver and interobserver variations^14^. Figure 3 shows examples of different methods used for ROI definition. All of these aspects have been evaluated in the determination of PET features in NSCLC patients (Table 1).

**Table 1:** Publications reporting methodological investigations on texture analysis in NSCLC patients.

Reference	Type of study	Patients, n	Setting, stage	Aspect evaluated	Lesion segmentation method	PET parameter and textural index matrix	Main results
Cheng^48^	R	56	Staging, I–III (only T)	Impact of respiration-averaged CT on PET texture parameters	Adaptive threshold, threshold uptake 45% of the SUV_max_^*^	FOS/IVH = 3 SS = 1 GLCM = 4 GLRLM = 3 NGTDM = 4	Texture parameters obtained with helical and respiration-averaged PET/CT showed a high degree of agreement (SUV entropy and entropy had the lowest levels of variation)
Cui^50^	n.r.	20	n.r.	Impact of the segmentation method on tumor volume estimation (validation of DM algorithm)	Automatic (DM), fuzzy C-means, threshold uptake 40% of the SUV_max_, threshold uptake 50% of the SUV_max_, tumor-customized downhill, watershed^§^	FOS/IVH = 1 NGTDM = 1 Gr = 1	DM algorithm was able to segment the tumor (also when adjacent to mediastinum or chest wall) and outperformed the other lung segmentation methods in terms of overlapping measure
Cui^51^	n.r.	40	n.r.	Impact of the segmentation method on tumor volume estimation (validation of topo-poly algorithm)	Threshold uptake 40% of the SUV_max_, threshold uptake 50% of the SUV_max_, adaptive threshold, fuzzy C-means, tumor-customized downhill, random walks, high-order interactive learning segmentation, PET/CT tumor-background likelihood model, topo-poly^§^	NGTDM = 1	Topo-poly algorithm was able to delineate tumor margins better than other methods
Dong^45^	R	50	Staging, I–IV	Impact of the segmentation method on tumor volume estimation	Absolute SUV cut-off of 2.5, manual (2 observers), threshold at 40% of the SUV_max_^*^	FOS/IVH = 1 SS = 1 GLCM = 1 + visual score	Intratumor heterogeneity significantly correlated with differences in the GTV definition (high heterogeneity corresponded to a larger GTV)
Gao^57^	n.r.	132	Staging, I–III	Impact of computer-based algorithm on diagnosis of mediastinal lymph node metastases (validation of computer-based algorithm)	Manual^#^	FOS/IVH = 3 GLCM = 5 + visual score	Diagnostic ability of computer-based algorithm and visual experience was similar
Hatt^44^	n.r.	25, only 17 analyzed	Staging, Ib–IIIb	Impact of the segmentation method on the tumor volume estimation	Adaptive threshold, fully automatic method (FLAB), manual, threshold at 50% of the maximum^*^	FOS/IVH = 1 SS = 1	All delineation methods except the manual one resulted in underevaluation of MTV. Anatomic tumor size and heterogeneity were correlated (larger lesions were more heterogeneous)
Hofheinz^49^	n.r.	30	n.r.	Impact of the segmentation method on tumor volume estimation (validation of voxel-specific threshold algorithm)	Lesion-specific threshold, manual, voxel-specific threshold^*°^	FOS/IVH = 2 SS = 1	Voxel-specific threshold method was able to reproduce tumor boundaries accurately, independent of the heterogeneity
Leijenaar^40^	n.r.	11 (test-retest cohort) + 23 (inter-observer cohort)		Features’ test–retest reliability and interobserver stability among multiple tumor delineation methods	Manual (by 5 observers), threshold at 50% of the maximum	FOS/IVH = 54 SS = 8 GLCM = 22 GLRLM = 11 GLSZM = 11	The majority of features had high test–retest (71%) and interobserver (91%) stability in terms of ICC
Leijenaar^52^	P	35	Staging, I–III	Comparison of different discretization methods for textural features	Manual (SUV discretization using a fixed bin size and a fixed number of bins)	GLCM = 22 GLRLM = 11 GLSZM = 11	SUV discretization had a crucial effect on textural features
Oliver^42^	R	23		Sensitivity of texture features to tumor motion by comparison of static (3D) and respiratory-gated (4D) PET imaging	Adaptive threshold (background-adapted thresholding method)^*^	FOS/IVH SS GLCM GLRLM (total 56)	Quantitative analysis using a 3D versus 4D acquisition provided notably different image feature values, mainly due to the impact of respiratory motion
Orlhac^46^	P	24	Staging, III	Impact of the segmentation method on the tumor volume estimation	Threshold at 40% of the maximum, adaptive threshold^*°^	FOS/IVH = 8 SS = 1 GLCM = 6 GLRLM = 11 GLSZM = 11 NGLDM = 3	IVH-based indices strongly depended on the tumor delineation method; 17/31 second- or high-order statistic features were robust with respect to tumor segmentation. Several texture indices included similar information. Some texture indices were highly correlated with MTV
Orlhac^53^	R	48	Staging, I–III	Impact of resampling step on textural features and on the ability of textural features to reflect tissue-specific patterns of metabolic activity	Adaptive threshold (relative resampling approach and absolute resampling approach)^*°^	FOS/IVH = 1 SS = 1 GLCM = 2 GLRLM = 3 GLSZM = 2	Textural features computed using an absolute resampling method varied as a function of the tissue type and cancer subtype more than when using the usual relative resampling approach
Tixier^55^	P	20	Staging, I–II	Impact of static and parametric acquisition on PET features	Fully automatic method (FLAB)^*°^	FOS/IVH = 2 SS = 3 GLCM = 3 GLSZM = 2	Compared with static SUV images, parametric images did not provide significant complementary information concerning heterogeneity quantification
van Velden^41^	P	11	Staging, IIIb–IV	Repeatability of texture features using different reconstruction settings and delineation methods	Threshold uptake 50% of the 3D SUV_peak_ on EANM-compliant (reconstruction method 1) and PSF-based (reconstruction method 2) images^°^	FOS/IVH = 29 FF = 3 SS = 10 GLRLM = 22 GLCM = 44 L = 1 SA = 2	The majority of features had a high level of repeatability (ICC ≥ 0.90 for 63 features). Features were more sensitive to a change in delineation method (n = 25) than a change in reconstruction method (n = 3)
Yan^47^	R	17	n.r., I–IV	Variability of PET textural features using different reconstruction methods, iteration numbers, and voxel size	Threshold uptake 40% of the SUV_max_^*°^	FOS/IVH = 6 GLCM = 21 GLRLM = 11 GLSZM = 13 NGLDM = 5 NGTDM = 5	Image features had different sensitivities to reconstruction settings (entropy_Hist_, difference entropy, inverse difference normalized, inverse difference moment normalized, low gray-level run emphasis, high gray-level run emphasis, and low gray-level zone emphasis were the most robust features; skewness, cluster shade, and zone percentage exhibited large variations)
Yip^56^	R	26	Staging, n.r.	Sensitivity of texture features to tumor motion by comparing static (3D) and respiratory-gated (4D) PET imaging	Threshold uptake 40% of the SUV_max_	GLCM = 1 GLRLM = 1 NGTDM = 4	4D-PET derived textures were less susceptible to tumor motion and may have greater prognostic value

FF: fractal features; FLAB: fuzzy locally adaptive Bayesian; FOS/IVH: first-order statistics/intensity-volume histogram; GLCM: gray-level co-occurrence matrix; GLRLM: gray-level run-length matrix; GLSZM: gray-level size-zone matrix; Gr: absolute gradient; ICC: intra-class correlation coefficient; L: Laplacian; LF: Laws family; n.a.: not available; n.r.: not reported; NGLDM: neighboring gray-level dependence matrix; NGTDM: neighborhood gray-tone difference matrix; P: prospective; R: retrospective; SA: spatial autocorrelation; SS: shape and size; W: wavelet

*Segmentation of only primary lung lesion.

^#^Segmentation of lymph nodes.

^§^ Segmentation of primary lung lesion and other tissues (e.g. lymph nodes).

°Included in the analysis only lung lesion with a volume > of a minimum cut-off (e.g. 3 mL).

Hatt *et al*.^44^ evaluated the impact of five different methods of segmentation on anatomic tumor volume, MTV, and heterogeneity (“coefficient of variation” histogram-based) in a small group of NSCLC patients. They found that all delineation methods except the manual one resulted in an underestimation of MTV, and that larger lesions were more heterogeneous.

Similar results were obtained in a larger population (n = 50) with NSCLC. Tumor volume was observed to be significantly diverse using different approaches (manual or automatic) on CT and on fused PET/CT images (volumes delineated on CT were larger than those defined on PET images). Intratumor heterogeneity, defined by visual scoring, “coefficient of variation”, or “entropy_GLCM_” (gray-level co-occurrence matrix – GLCM) significantly correlated with differences in the target volume [tumors with a high heterogeneity showed a larger gross tumor volume (GTV)], suggesting that caution should be exercised when applying relatively simple threshold-based segmentation to define the target volume for tumors with high heterogeneity^45^.

Hofheinz *et al*.^49^ developed and tested a voxel-specific threshold algorithm as a delineation method for heterogeneous tumors. This method, which can be considered as an extension of an adaptive threshold method, proved able to reproduce the true tumor boundaries accurately, without being influenced by the heterogeneity (“coefficient of variation”).

Cui *et al*.^50^ developed an automatic algorithm that used the PET SUV volume and the CT volume to localize and segment tumor lesions. This algorithm outperformed other (semi-)automatic methods in terms of overlapping measure, and they found that the feature “contrast_NGTDM_” (NGTDM: neighbor gray-tone difference matrix based) was valuable in automatic tumor localization. The same group developed and tested a “topo-poly” algorithm (which incorporated an intensity graph and a topology graph) in two groups of patients defined as having ‘isolated’ (i.e., lung tumor located in the lung parenchyma and away from associated structures/tissues in the thorax) or ‘complex’ (i.e., tumor abutted/involving a variety of adjacent structures, where the tumor margins were indistinct and/or had heterogeneous regions of FDG uptake) disease. This method provided better anatomic and functional boundary delineations for both small and large tumors and for ‘complex’ cases. Again, “contrast_NGTDM_” was valuable in automatic tumor localization^51^.

Leijenaar *et al*.^40^ tested more than 100 PET features (first-order statistics and intensity volume histogram – FOS/IVH, “shape and size” features – SS, GLCM, gray-level run-length matrix – GLRLM, and gray-level size-zone matrix – GLSZM) to evaluate their test–retest reliability and interobserver stability among different tumor delineation methods in 34 NSCLC patients. Considering all features, a good overall similarity in feature stability was observed, based on rankings in terms of test–retest and interobserver intra-class correlation coefficient (ICC, p ≪ 0.001). Comparing stability rankings per feature group, a high similarity was found for both the first-order statistics (p ≪ 0.001) and other textural features (p ≪ 0.001). Features based on GLSZM had the overall lowest ranks, indicating that these features have the highest variability. For the IVH features the observed similarity was more moderate (p ≪ 0.001). Comparison of the rankings for the geometric features proved non-significant (p = 0.086). Overall, more stable features on repeated PET scans were also more robust against interobserver variability. In a similar number of patients, the same group evaluated prospectively different discretization methods (fixed bin size *versus* fixed number of bins) for textural feature extraction in the context of treatment response assessment. Textural feature values were shown to depend on the intensity resolution used for SUV discretization. Discretizing SUVs using a fixed number of bins was found to be less appropriate for inter- and intrapatient comparison of textural feature values in a clinical setting. Additionally, results obtained for the features could not be directly compared when different intensity resolutions were used, suggesting that their interpretation (e.g., prognostic or predictive value) depended on the intensity resolution. It is noteworthy that the “correlation_GLCM_” was the only feature observed to have highly similar patient rankings over the course of treatment, regardless of the discretization method or discretization value used^52^.

Orlhac *et al*.^46^ investigated a consistent number of texture indices on a variety of tumors (including 24 NSCLC) to gain a better insight into how they relate to one another and to conventional indices such as SUV, MTV, and TLG and to determine the extent of their robustness with respect to the gray-level resampling scheme and formula and to the tumor delineation method. All histogram indices strongly depended on the tumor delineation method. Similarly, “contrast_NGTDM_”, “busyness” (NGTDM-based), “low gray-level run emphasis”, “short-run low gray-level emphasis”, “long-run low gray-level emphasis” (GLRLM-based), “low gray-level zone emphasis”, and “short-zone low gray-level emphasis” (GLSZM-based) were highly sensitive to the segmentation method, while “homogeneity_GLCM_” and “entropy_GLCM_” were found to be robust with respect to tumor segmentation. The same group investigated, in 48 treatment-naïve NSCLC patients, the effect of the resampling approach on the ability of textural features to reflect tissue-specific patterns of metabolic activity. An adaptive threshold method was used to delineate tumors. The relative resampling approach (RR) was compared with the absolute resampling (AR) approach. Seven features (from GLCM, GLRLM, and GLSZM) were calculated and correlated with tissue types and cancer subtypes. AR-based “entropy_GLCM_” could differentiate between tumor and healthy tissue (p < 0.0001). Using the AR method, tumor tissue exhibited higher “high gray-level zone emphasis” than healthy tissue, while tumors had lower “homogeneity_GLCM_” and “low gray-level zone emphasis”. AR-based textural features differed adenocarcinoma (Adk) and squamocellular carcinoma (Sqc) (p ≤ 0.05)^53^.

Yan *et al*.^47^ tested the variability of more than 60 PET-textural features using different reconstruction settings, different iteration numbers, and different voxel size in 17 NSCLC patients. “Skewness” (IVH-based), “cluster shade_GLCM_”, and “zone percentage” (GLSZM-based) were the least robust with respect to reconstruction algorithms using default settings and were the most sensitive to iteration number. Among all the features evaluated, “entropy_Hist_”, “difference entropy”, “inverse difference normalized”, “inverse difference moment normalized”, “low gray-level run emphasis”, “high gray-level run emphasis”, and “low gray-level zone emphasis” proved to be the most robust.

Recently, repeatability of more than 100 radiomics features using different reconstruction settings, first using the point spread function and secondly complying with the European Association of Nuclear Medicine (EANM) guidelines for tumor PET imaging^54^, and using different delineation methods, first on PET and then on CT images, was evaluated in 11 NSCLC patients. The best performance was seen using CT-based delineation (32%), followed by EANM-compliant reconstruction (17%), PET-based delineation (17%), and point spread function-based reconstruction (10%). The majority of PET features (98%) had a repeatability comparable to that reported for simple SUV measures (e.g., SUV_max_) in the literature. Sixty-three features showed a very high ICC (≥0.90) independent of delineation or reconstruction. The performance of radiomics features depended more on the delineation method than on the applied reconstruction algorithm (changes in 25 and 3 features, respectively). CT-based delineation showed favorable repeatabilities and ICCs for most radiomics features, an exception being shape-based features, for which PET-based delineation performed better^41^.

Compared with static images, parametric images don’t provide significant complementary information concerning standard parameters (SUV_max_, SUV_mean_, and metabolically active tumor volume - MATV) and heterogeneity quantification (histogram-based)^55^. Differences in quantitative analysis using three-dimensional (3D) versus respiratory-gated (4D) acquisition have been reported. According to Oliver *et al*.^42^, the features with the least variability were “sphericity”, “spherical disproportion”, “entropy_Hist_”, “entropy_GLCM_”, “sum entropy”, “information measure of correlation 2”, “short run emphasis”, “long run emphasis”, and “run percentage”, while the features with the largest differences (>50%) were “kurtosis”, “low gray-level run emphasis”, “short run low gray-level emphasis”, and “long run low gray-level emphasis”.

Yip *et al*.^56^ found significant differences in “maximal correlation coefficient”, “long run low gray-level emphasis”, “coarseness”, and “busyness” (NGTDM-based) between 3D and 4D PET imaging. When measuring tumor heterogeneity characteristics, reduced motion blurring by 4D PET acquisition was found to offer significantly better spatial resolution of textural features. 3D PET textures may lead to inaccurate prediction of treatment outcome, hindering optimal management of lung cancer patients. 4D PET textures may have a better prognostic value as they are less susceptible to tumor motion^42, 56^. Different results have been reported by Cheng *et al*.^48^, who compared the attenuation correction of PET images with helical CT (PET/HCT) and respiration-averaged CT (PET/ACT) in 56 NSCLC patients. PET/ACT yielded significantly higher SUV_max_, SUV_mean_, and TLG while significant differences between PET/HCT and PET/ACT were not observed with regard to other features, including “entropy_Hist_”, “entropy_GLCM_”, “dissimilarity”, “homogeneity_GLCM_”, and “uniformity” (GLCM-based), “gray-level non-uniformity”, “zone-size non-uniformity”, and “high gray-level large zone emphasis” (GLSZM-based), and “coarseness”, “busyness”, “contrast_NGTDM_”, and “complexity” (NGTDM-based).

Textural features have also been used to develop a computer-based algorithm which supported a vector machine; combined image parameters, derived from CT, PET, and PET/CT images, were found to improve diagnosis of mediastinal lymph node metastases by PET/CT^57^.

### Texture analysis and clinical applications

#### Diagnosis

Imaging texture analysis has been evaluated in order to determine which type and level of tissue heterogeneity can be captured and quantified through PET and to bridge the gap between *in vivo* and *ex vivo* tumor characterization^58^. Histologic characteristics and PET features have been compared to identify whether texture analysis can help in differentiating between benign and malignant lesions or in classifying NSCLC subtypes (Table 2).

**Table 2:** Publications reporting studies on the diagnostic, prognostic and predictive role of texture analysis in NSCLC patients.

Reference	Type of study	Patients, n	Setting, stage	Aspect investigated	Lesion segmentation method	PET features and textural index matrix	Main results
Apostolova^73^	R	60	Staging, I–III	Prognostic value of asphericity	Adaptive threshold method^*°^	FOS/IVH = 2SS = 4	Asphericity was a predictor of progression-free survival and overall survival
Budiawan^59^	R	44	Staging, I–IV	Ability of PET features to predict lymph node metastases	Manual^#°^	FOS/IVH = 4 + visual score	Metastatic lymph nodes had higher heterogeneity (coefficient of variation) than inflammatory ones
Carvalho^80^	n.r.	220	Staging, I–IIIb	Prognostic value of heterogeneity based on PET textural features	Absolute SUV cut-off values of 2.5, 3, and 4, threshold at 40% and 50% of SUV_max_	FOS/IVH = 8 SS = 1	Best prognostic value for overall survival was found for relative portions of the tumor above higher uptakes (80% SUV)
Cook^77^	R	53	Staging, I–III	Ability of PET features to predict prognosis and disease progression after concurrent chemoradiotherapy	Threshold at 45% of the SUV_max_^*^	FOS/IVH = 3 SS = 2 NGTDM = 4	Coarseness, contrast, and busyness were associated with response to chemoradiotherapy and prognosis
Cook^78^	P	47	Staging, IIIb–IV	Ability of PET features to predict prognosis and disease progression after erlotinib	Threshold at 40% of the SUV_max_^*^	FOS/IVH = 8 SS = 2 NGTDM = 4	Heterogeneity predictedresponse to erlotinib. Changes in entropy_Hist_ (baseline and 6 weeks) were independently associated with overall survival and treatment response
Desseroit^83^	R	116	Staging, I–III	Develop a nomogram by exploiting intratumor heterogeneity (PET and CT features) to identify patients with the poorest prognosis	Fully automatic method (FLAB)	FOS/IVH = 3 SS = 1 GLCM = 2 GLSZM = 2 ( + 35 on CT images)	Intratumor heterogeneity could be used to create a nomogram with a higher stratification power than staging alone (poorest prognosis: stage III, large tumor volume, high PET heterogeneity, and low CT heterogeneity)
Fried^81^	R	195	Staging, III	Ability of PET features to enhance overall survival risk stratification	Manual^§°^	FOS/IVH = 8 SS = 3 GLCM = 4	Imaging features (solidity and primary tumor energy) improved risk stratification
Fried^82^	R	225	Staging, III	Ability of PET features to identify patients who might benefit from a higher radiation dose compared with that for the entire stage III	Semiautomatic gradient based^§^	FOS/IVH = 1 SS = 3 GLCM = 1	Imaging features were found to be capable of isolating subgroups of patients who received a benefit or detriment from dose escalation
Ha^60^	R	30	Diagnostic, n.r.	Correlation between metabolic heterogeneity and histopathologic characteristics	Adaptive threshold^*^	FOS/IVH = 1 GLCM = 21 Gr = 2	The majority of texture features analyzed (including SUV_max_) differed between Adk and Sqc
Hatt^74^	R	101	Staging, I–III	Relationship between tumor MTV and derived heterogeneity measurements	Fully automatic method (FLAB)^*°^	FOS/IVH = 3 SS = 1 GLCM = 2 GLSZM = 2	Correlation between MTV and textural features varied greatly depending on the MTV (reduced correlation for increasing volumes)
Kang^75^	R	116	Staging, III	Ability of PET features to predict disease progression after concurrent chemoradiotherapy	Absolute SUV cut-off value of 3.0^*^	FOS/IVH = 2 SS = 1	Intratumoral heterogeneity predicted disease progression after chemoradiotherapy in inoperable stage III NSCLC
Kim^61^	R	119	Staging, I	Ability of PET features to predict prognosis after curative surgical resection in pathologically N0 tumor	Absolute SUV cut-off value of 2.5^*^	FOS/IVH = 2 SS = 2	Heterogeneity of primary tumor was predictive of recurrence in pN0 Adk but not in Sqc
Lovinfosse^36^	R	63	Staging, I	Ability of PET features to predict prognosis after radiotherapy	Fully automatic method (FLAB)^*^	FOS/IVH = 7 SS = 2 GLCM = 6 GLSZM = 2 NGTDM = 3	Intratumoral heterogeneity (dissimilarity) appeared to be a strong independent outcome predictor after radiotherapy
Miwa^37^	R	54	Diagnostic, n.a.	Ability of PET and CT features to differentiate malignant from benign pulmonary nodules	Threshold at 40–100% (intervals of 2%) of SUV_max_^*^	FOS/IVH = 1 FF = 1 (+1 on CT images)	Intratumoral heterogeneity could help to differentiate malignant and benign pulmonary nodules (better diagnostic ability of density fractal dimension on PET than morphological fractal dimension on CT)
Nair^71^	R	172 (study cohort = 25, external cohort = 63, validation cohort = 84)	Staging, I–IV (study cohort) and I–II (validation cohort)	Identify individual genes and gene expression signatures associated with prognostically relevant PET features	Adaptive threshold method^*^	FOS/IVH = 10 SS = 3	Four genes (LY6E, RNF149, MCM6, FAP) associated with textural features were also associated with survival
Ohri^76^	P	250, only 201 analyzed	Staging, IIb–III	Prognostic value of heterogeneity based on PET textural features	Semiautomatic gradient-based	FOS/IVH SS GLCM GLRLM GLSZM NGTDM NGLDM (total 45) + visual score	SumAverg was an independent predictor of overall survival
Pyka^79^	R	45	Staging, I	Ability of PET features to predict prognosis and disease progression after radiotherapy	Absolute SUV cut-off values of 2.0 and 2.5^*^	FOS/IVH = 3 SS = 1 GLCM = 2 NGTDM = 3	Tumor heterogeneity was associated with response to radiation therapy
Tixier^35^	R	108, only 102 analyzed	Staging, I–III	Prognostic value of heterogeneity	Fully automatic method (FLAB)^*^°^	FOS/IVH = 3 SS = 2 GLCM = 3 GLSZM = 3 + visual score	High SUV, large metabolic volumes, and high heterogeneity were associated with poorer overall survival and recurrence-free survival
Vaidya^29^	R	27	Staging, I–IV	Ability of PET and CT features to predict disease progression after radiotherapy	Manual	FOS/IVH = 12 SS = 2 GLCM = 4 ( + 32 on CT images)	IVH parameters (I_x_ metrics for PET and V_x_ metrics for CT) yielded the highest association with locoregional control
van Gómez López^62^	R	38	Staging, I–IIIa	Correlation between metabolic heterogeneity and pathologic staging	Absolute SUV cut-off value of 2.5^*^	FOS/IVH = 2 SS = 2 GLCM = 5	Tumor heterogeneity was correlated with global metabolic parameters, and both were associated with macroscopic tumor diameter and, under special conditions (exclusion of a small tumor with high AJCC stage), with the AJCC stage
Win^13^	P	122 (study cohort = 56, validation cohort = 66)	Staging, I–IV	Ability of PET and CT features to predict survival	Threshold at 42% of the SUV_max_^*^	FOS/IVH = 2 ( + 1 on CT images)	PET-derived heterogeneity was predictive of survival at univariate analysis; at multivariate analysis only CT-derived heterogeneity, stage, and permeability were independent predictors of survival
Wu^84^	R	101 (study cohort = 70, validation cohort = 31)	Staging, I	Ability of PET features to predict distant metastases	Fully automatic method^*^	FOS/IVH = 11 SS = 2 GLCM = 3 W = 24 LF = 30	The optimal prognostic model for identifying groups at risk of developing distant metastasis included SUV_peak2mL_ and Gauss cluster shade_Laws_

Adk: adenocarcinoma type; FF: fractal features; FLAB: fuzzy locally adaptive Bayesian; FOS/IVH: first-order statistics/intensity-volume histogram; GLCM: gray-level co-occurrence matrix; GLRLM: gray-level run-length matrix; GLSZM: gray-level size-zone matrix; Gr: absolute gradient; ICC: intra-class correlation coefficient; L: Laplacian; LF: Laws family; n.a.: not available; n.r.: not reported; NGLDM: neighboring gray-level dependence matrix; NGTDM: neighborhood gray-tone difference matrix; P: prospective; R: retrospective; SA: spatial autocorrelation; Sqc: squamocellular types; SS: shape and size; W: wavelet.

*Segmentation of only primary lung lesion.

^#^Segmentation of lymph nodes.

^§^Segmentation of primary lung lesion and other tissues (e.g. lymph nodes).

^Application of partial volume correction.

°Included in the analysis only lung lesion with a volume > of a minimum cut-off (e.g. 3 mL).

As mentioned above, Orlach *et al*.^53^ compared the relative with the absolute resampling approach (RR and AR, respectively) and calculated the correlations of seven features with tissue types (tumor versus healthy tissue) and cancer subtypes (Adk versus Sqc). RR-based “entropy_GLCM_” didn’t distinguish between tumor and healthy tissue (p = 0.7621) whereas the same index computed with the AR method was able to differentiate between these tissue types (p < 0.0001). Using the AR method, tumor tissue exhibited higher “high gray-level zone emphasis” than healthy tissue, while tumors had lower “homogeneity_GLCM_” and “low gray-level zone emphasis”. Comparing textural indices in Adk versus Sqc, all RR-based textural features were not significant (p > 0.07), in contrast to the AR-based textural features (p ≤ 0.05). According to these results, features computed using an AR method vary as a function of the tissue type and cancer subtype and might be useful for tumor characterization.

Miwa *et al*.^37^ evaluated whether morphological complexity (“morphological fractal dimension” derived from CT) and intratumoral heterogeneity (“density fractal dimension” derived from PET) assessed by fractal analysis improved the differential diagnosis between benign and malignant lung nodules in 54 patients with suspected NSCLC. Both fractal dimensions assessed by PET and CT were lower in malignant than in benign nodules (p < 0.05). SUV_max_ was higher in malignant than in benign nodules (p < 0.05). Tumor size significantly correlated with SUV_max_ (p < 0.0001), but not with either “morphological fractal dimension” (p = 0.61) or “density fractal dimension” (p = 0.09). The diagnostic accuracy of “density fractal dimension” tended to be higher than SUV_max_ (78% versus 68%, respectively) and was better than that for “morphological fractal dimension” (65%).

Heterogeneity has also been evaluated to determine whether it can help in differentiating between metastatic and inflammatory lymph nodes in lung Adk, as assessed by visual analysis, and other standard parameters of PET and CT (size and Hounsfield units). In this study, heterogeneity was assesed as “coefficient of variation” of lymph nodes in 44 patients (with a total of 94 biopsy-proven lymph nodes). Visual assessment for malignancy had high sensitivity (81%) but a relatively low specificity (67%), with an accuracy of 75%. The diagnostic performance of PET/CT using the cut-offs commonly employed for standard PET and CT parameters (SUV_max_ = 2.5, size of lymph nodes = 1 cm, and Hounsfield units = 120) was not satisfactory (accuracy of 56%, 60%, and 68%, respectively). Using an optimal cut-off determined by this study (SUV_max_ = 5.96, size of lymph nodes = 1.5 cm, and Hounsfield units = 136), the accuracy increased for SUV_max_ and size but not for Hounsfield units (81%, 84%, and 65%, respectively). Heterogeneity measured as “coefficient of variation” (using a cut-off = 0.2) yielded good sensitivity, specificity, and accuracy (88%, 76%, and 82%, respectively). The accuracy of “coefficient of variation” was slightly higher than that of SUV_max_ and size when using optimal cut-offs, but significantly higher than that of visual assessment and Hounsfield units. “Coefficient of variation”, SUV_max_ and size were significantly higher in metastatic lymph nodes than in benign ones (p < 0.0001), while the Hounsfield unit value was significantly lower in metastatic than in benign lymph nodes (p = 0.0249). Univariate analysis showed that all parameters except visual assessment were significant predictors, while using multivariate logistic regression only “coefficient of variation” and size proved statistically significant (p = 0.032 and 0.023, respectively)^59^.

Yip *et al*.^56^, in order to evaluate whether texture features may be affected differently in Adk (21 lesions) versus Sqc (13 lesions) by motion, calculated the relative difference in each texture between 3D and 4D PET. The relative difference in each texture between 3D and 4D PET was not found to be significantly different between histologies (p = 0.26).

Ha *et al*.^60^ analyzed differences in 24 textural features between Adk and Sqc (17 and 13 patients, respectively). The majority of texture parameters that showed a significant difference between Adk and Sqc were derived from GLCM (93%). SUV_max_ showed the most significant association with tumor pathology (p = 0.001). Upon autoclustering by linear discriminant analysis with those texture parameters that showed a significant difference between tumor subtypes (n = 15), the classification accuracy was found to be 83% (25/30 lesions were correctly clustered to their own tumor subtype). When analyzing with all parameters (n = 24), linear discriminant analysis clustered the lesions accurately according to their pathology, i.e., Adk versus Sqc, with a classification accuracy of 100% (linear separability of this autoclustering = 0.90).

Similarly, Kim *et al*.^61^ found that SUV_max_, MTV, TLG, and heterogeneity (defined as the derivative of the volume-threshold function from 20 to 80%), were significantly higher in Sqc than in Adk.

van Gómez López *et al*.^62^ evaluated the correlation between conventional metabolic parameters (SUV_max_, SUV_mean_, MTV, and TLG) and heterogeneity (“energy_GLCM_”, “contrast_GLCM_”, “correlation”, “entropy_GLCM_”, and “homogeneity_GLCM_”), histology, tumor size, and AJCC stage in 38 NSCLC patients (24 Sqc and 14 Adk). There was a positive relationship for all metabolic parameters with “entropy_GLCM_”, “correlation”, and “homogeneity_GLCM_” and a negative relationship with “energy_GLCM_” and “contrast_GLCM_”. No statistically significant differences were found between the mean values of tumor size, AJCC stage, and standard metabolic parameters in Adk versus Sqc tumors. Concerning textural features, “energy_GLCM_” was lower in Adk than in Sqc (p = 0.027) while “homogeneity_GLCM_” was higher in Adk than in Sqc (p = 0.047). Tumor size was correlated with “energy_GLCM_”, “contrast_GLCM_”, “correlation”, “entropy_GLCM_”, “MTV”, and “TLG” (p < 0.01). A statistical correlation between the pT and “energy_GLCM_”, “contrast_GLCM_”, “entropy_GLCM_”, and “MTV” (p ≤ 0.05) was found, but not between remaining AJCC subgroups and the other textural or metabolic parameters.

#### Prognosis and treatment response prediction

The Warburg effect, first described over 80 years ago, postulates that tumors undergo glycolysis preferentially despite adequate intracellular oxygen tension^63, 64^. While Warburg believed this to be a consequence of mitochondrial dysfunction, tumor glycolysis can proceed with functional cellular mitochondria and may be an adaptive response for tumor survival^65–67^. Furthermore, studies have recently linked glycolysis in cancer to more widespread deregulation of cell bioenergetics^68–70^, suggesting that FDG uptake may be a surrogate for more than glycolysis alone and perhaps a lens through which one can view global tumor bioenergetics^71^. Therefore, texture features have been introduced as imaging biomarkers on the assumption that they are an index of the degree of tumor heterogeneity, and that biologic tumor heterogeneity is associated with poor prognosis in cancer patients and can contribute to treatment failure and drug resistance^72^. The prognostic value of texture analysis has been evaluated in different NSCLC clinical settings (Table 2).

Nair *et al*.^71^ evaluated a possible association between textural features, gene expression signatures, and survival in a computational study (172 NSCLC patients). Fourteen PET features were extracted within the study cohort (n = 25). Individual genes associated with PET features in the study cohort were directly analyzed in the external cohort (n = 63) for their association with clinical outcomes. Lastly, PET features associated with prognostic gene signatures from the external cohort were tested in a validation cohort (n = 84). Four genes (LY6E, RNF149, MCM6, FAP) associated with textural features were found also to be associated with survival. Histogram-based and “shape and size” features together provided a more accurate prognostic model than each feature alone, suggesting that leveraging tumor genomics with an expanded collection of PET features may enhance understanding of the value of FDG uptake as an imaging biomarker beyond its association with glycolysis.

Win *et al*.^13^ compared the prognostic value of texture analysis with tumor staging and other imaging prognostic factors (i.e., metabolism assessed by PET/CT and permeability assessed by dynamic contrast-enhanced CT) in 122 NSCLC patients treated with curative or palliative approach. Tumor heterogeneity (“entropy_Hist_”) was calculated from both attenuation-corrected CT images and SUV images without image filtration. “Entropy_Hist_” (derived from both CT and PET images), permeability, and stage were found to be survival predictors at univariate analysis (p ≤ 0.003), in contrast to SUV_max_ (p = 0.948). At multivariate analysis, “entropy_Hist_” derived from CT (p = 0.021), stage (p = 0.001), and permeability (p < 0.001) were identified as independent survival predictors, irrespective of the treatment objective (curative or palliative). In the study by Cheng *et al*., “Entropy_Hist_”, “entropy_GLCM_”, and “coarseness”, derived from both PET/HCT and PET/ACT, were able to predict disease-specific survival at univariate (p ≤ 0.01) and multivariate analysis (p < 0.05) in stage I–III NSCLC patients^48^.

Similarly, the “shape and size” features “asphericity” (p < 0.001) and “solidity” (p = 0.05), as well as “primary surgical treatment” (p = 0.05), were found to be significant independent predictors of progression-free survival in 60 NSCLC patients treated with different approaches. Concerning overall survival, only “asphericity” and “primary surgical treatment” (p = 0.02 and = 0.01, respectively) proved to be independent predictors, and none of the other PET parameters (including SUV_max_, TLG, and MTV) showed a significant predictive value in this series of patients^73^.

The GLCM- and GLSZM-derived features “entropy_GLCM_”, “homogeneity_GLCM_”, “dissimilarity”, “size-zone variability”, and “zone percentage”, but not “high intensity emphasis” (GLSZM-based), have also been reported to be independent prognostic factors with respect to stage (although not independently of each other) in patients treated with different approaches. Nonetheless, the addition of risk factors allowed a better differentiation of patient outcome. High SUV, large metabolic volumes, and high heterogeneity were associated with a poorer overall survival and recurrence-free survival^35^, suggesting that heterogeneity quantification and volume (i.e., MTV) may provide valuable complementary information with respect to prognosis, although the complementary information increases substantially with larger volumes^74^.

Heterogeneity of primary tumor (evaluated by the area under the curve of cumulative SUV histograms: AUC-CSH) was observed to be an independent predictor of recurrence in pathologically N0 Adk but not in Sqc (p = 0.03 and 0.13, respectively) after curative surgical resection^61^ as well as a predictor of disease progression after concurrent chemoradiotherapy in patients with inoperable stage III NSCLC^75^.

Interestingly, “Sum Average” (GLCM-based) was strongly associated with overall survival in a multi-institutional dataset of locally advanced NSCLC patients with large tumors who were treated with definitive chemoradiotherapy, suggesting its robustness as a prognostic factor^76^.

NGTDM-derived features (“coarseness”, “contrast_NGTDM_”, and “busyness”) have also been reported to be associated with response to chemoradiotherapy and prognosis^77^ in locally advanced NSCLC. The same group, testing FOS and high-order features as predictors of response or survival in patients treated with erlotinib, found that response to erlotinib was associated with reduced heterogeneity and that the percentage of changes in “entropy_Hist_” (between baseline and 6-week PET/CT) was independently associated with overall survival and treatment response^78^.

Similarly, PET features have been reported to be able to predict outcome and/or treatment response in NSCLC patients treated with definitive radiotherapy^29, 36, 79, 80^. In this specific clinical setting, “entropy_GLCM_” has been reported to be an independent predictor of disease-specific survival (p = 0.016)^79^, while “dissimilarity” has been found to be associated with both disease-specific survival (p = 0.037) and disease-free survival (p < 0.01)^36^.

Initial attempts have been made to determine whether quantitative imaging features from pretreatment PET can enhance overall survival risk stratification beyond what can be achieved with conventional prognostic factors in NSCLC. In patients with stage III NSCLC, linear predictors of overall survival generated with both quantitative imaging features (histogram-derived, GLCM-derived, and “shape and size” features) and conventional prognostic factors (age, sex, histologic findings and stage, Karnofsky performance status, smoking status and estimated pack-years, treatment type) have demonstrated improved risk stratification compared with those generated with conventional prognostic factors alone in terms of log-rank statistics (p = 0.18 versus = 0.0001, respectively)^80^. The use of quantitative imaging features selected during cross-validation improved the model using conventional prognostic factors alone (p = 0.007). Disease “solidity” and primary tumor “energy_GLCM_” were found to be selected in all folds of cross-validation^81^. Additionally, these features were found to be capable of isolating subgroups of patients who received a benefit or detriment from dose escalation^82^.

Similarly, PET “entropy_GLCM_” and CT “zone percentage” have been found to have the highest complementary values with clinical stage and functional volume in stage I–III NSCLC^83^. Desseroit *et al*.^83^ provided a nomogram able to improve stratification amongst patients with stage II and III disease, allowing identification of those with the poorest prognosis (clinical stage III, large MTV, high PET heterogeneity, and low CT heterogeneity).

In early-stage NSCLC the optimal prognostic model for prediction of distant metastases in patients treated with stereotactic ablative radiation therapy included two image features that allowed quantification of intratumor heterogeneity and SUV_peak_. A significant improvement (p = 0.0001) in predicting freedom from distant metastasis was seen when histologic information was added compared with a prognostic model based solely on imaging features^84^.

## Discussion

Texture features are of growing interest for tumor characterization in imaging. Nevertheless, on the basis of results published to date on FDG PET, it is unclear which indices should be used, what they represent, and how they are related to conventional parameters such as SUVs, MTV, and TLG^46^. We summarize the results of the available studies within Table 3. PET features differed significantly in malignant and non-malignant tissues (considering either primary lung tumors or lymph nodes)^37, 46, 59^ and also in Adk and Sqc^60–62^. However, literature data are really heterogeneous in this setting and, despite promising results, it isn’t possible to suggest for use a reproducible feature or a combination of features able to characterize definitely malignant tissues or lung cancer subtypes.

**Table 3:** Summary of clinically relevant results in investigations assessing the diagnostic, prognostic and predictive role of FDG-PET/CT texture analysis.

**Diagnostic role**
Compared with non-malignant lesions, malignant lung nodules are characterized by higher SUV_max_ and lower morphological and density fractal dimensions^37^.
Metastatic lymph nodes are characterized by higher heterogeneity (coefficient of variation) than inflammatory ones^59^.
Large lesions are characterized by high heterogeneity (i.e., visual score, entropy_GLCM_, coefficient of variation)^44, 45, 74^.
Each subtype of NSCLC tumor has different metabolic heterogeneity characteristics. Compared with Adk, Sqc is characterized by higher SUV_max_, AUC-IVH, energy_GLMC_, entropy_GLCM_, sum entropy, difference entropy, and inverse different moment and by lower homogeneity_GLCM_, sum of squares, angular second moment, ratio of non-zero_Gr_, and difference variance^60–62^.
**Prognostic and predictive role**
Heterogeneity (i.e., AUC-CSH) can predict recurrence in pN0 Adk patients who have undergone curative surgery but not in Sqk patients (high heterogeneity is associated with a shorter DFS)^61^.
Best prognostic value for overall survival is found for relative portions of the tumor above higher uptakes defined as SUV_max_ > 80% (i.e., V_80_) in patients who received radiation therapy (sequential chemoradiation, concurrent chemoradiation, or only radiation). The higher the portion above higher uptake (V_80_), the better the prognosis^29, 80^.
Heterogeneity (i.e., low AUC-CSH) identifies patients with inoperable stage III NSCLC with poor PFS^75^.
High SUV_max_, large MTV, and high heterogeneity (i.e., high entropy_GLCM_, high asphericity, homogeneity_GLCM_, and high dissimilarity, size-zone variability, and low zone percentage) are associated with poorer OS and RFS in stage I–III NSCLC^35, 73, 74, 83^.
Tumor heterogeneity (i.e., entropy_GLCM_) is associated with response to radiation therapy in NSCLC (DSS is lower for patients with high entropy_GLCM_)^79^.
Lesions in responders (complete or partial response) to chemoradiotherapy are characterized by lower coarseness, contrast_NGTDM_, and busyness than non-responders (stable or progressive disease). High coarseness values are associated with an increased risk of progression (increased risk of death), whereas high contrast_NGTDM_ and busyness values are associated with a lower risk of progression (PFS and LPFS)^14^.
Large primary tumors with low SumAverage (i.e., more heterogeneous) have a poor prognosis following chemoradiotherapy^76^.
Lesions in responders to erlotinib are characterized by lower heterogeneity than those in non-responders. Specifically, lower heterogeneity after 6 weeks of treatment, as measured by contrast _NGTDM_, is independently associated with longer survival, and a larger reduction in heterogeneity between baseline and 6 weeks of treatment, as measured by entropy_Hist_, is independently associated with longer survival and with treatment response^78^.
Tumor heterogeneity (i.e., dissimilarity) appears to be a strong independent outcome predictor (DSS and DFS) after radiotherapy. Low dissimilarity is associated with a higher risk of recurrence^36^.
The optimal prognostic model for identification of groups of NSCLC patients at risk for developing distant metastasis includes SUV_peak2mL_ and Gauss cluster shade_Laws._ High SUV_peak2mL_ and Gauss cluster shade_Laws_ are associated with an increased risk of distant metastases^84^.
Solidity (which quantifies the dispersion of primary and nodal disease in a local region, with high values corresponding to disease that is compact and in close proximity, and low values corresponding to disease that is dispersed) and primary tumor energy_GLCM_ (higher level for tumors that are more heterogeneous) improve risk stratification compared with a model with conventional prognostic factors alone in stage III NSCLC. Solidity and primary tumor energy_GLCM_ are capable of isolating subgroups of patients who will receive a benefit or detriment from dose escalation (i.e., as disease solidity and primary co-occurrence matrix energy increase, patients receiving higher dose radiation therapy have improved OS and PFS compared with those receiving lower doses)^81, 82^.

Adk: adenocarcinoma type; AUC-IVH: area under the curve within the intensity volume histogram; DFS: disease-free survival; DSS: disease-specific survival; GLCM: gray-level co-occurrence matrix; GLRLM: gray-level run-length matrix; GLSZM: gray-level size-zone matrix; Gr: absolute gradient; LPFS: local progression-free survival; MTV: metabolic tumor volume; NGTDM: neighborhood gray-tone difference matrix; NSCLC: non-small cell lung cancer; OS: overall survival; PFS: progression-free survival; Sqc: squamocellular types; SUV: standardized uptake value.

Again, different metrics, matrices, and methods (e.g., tumor segmentation, survival endpoints) have been reported in the evaluation of NSCLC prognosis based on PET features. Commonly, the term “heterogeneous” is used with different meanings. Concerning texture analysis, “heterogeneity” may result from one or more PET features positively or negatively related to treatment and/or outcome. Therefore clinical texture papers often use the term “heterogeneity” to summarize specific tumor characteristics expressed by PET features. Among these, “entropy_Hist_” and “entropy_GLCM_” are most frequently reported to have an independent prognostic role, able to predict treatment outcome and/or survival in NSCLC patients^13, 29, 35, 48, 78, 79, 83^.

It is important for textural feature values to be directly comparable, both between and within patients, in order to derive meaningful conclusions from radiomic analysis and allow their use in clinical routine. However, the lack of a standardized method to calculate textural features prevents comparison between literature data and meta-analysis. Additionally, crucial information about texture extraction is not always available within the published articles. In order to try to simplify the complexity of texture analysis and to facilitate comparison among different series, some mandatory information concerning specific methodological aspects should be reported. Specifically, in the drafting of a paper on PET texture analysis, the following should be indicated in the Methods section (Table 4). Specifically, in the drafting of a paper on PET texture analysis, the following should be indicated in the Methods section: (a) the scanner, (b) the method (e.g., respiratory motion, dynamic) and parameters used to (c) acquire and (d) reconstruct images, (e) the type of images used to extract features (i.e., PET or both PET and CT), (f) the “target” of texture analysis (e.g., primary tumor, lymph nodes), (g) the application of PVC and/or a minimum lesion size/volume, (h) the method of segmentation (e.g., threshold uptake 40% of the SUV_max_), (i) the discretization method (e.g., fixed number of bins), (j) the software, and (k) the feature(s) and matrix computation method. As the published results are very preliminary, a preferred method for reconstruction, discretization, or segmentation cannot be recommended. Obviously, consistent terminology in respect of features (a proposal is reported within the Supplementary material) and an appropriate statistical analysis are mandatory. Datasets of 10–15 patients per feature have been recommended to test the prognostic power of texture features. Moreover, use of the radiomics features insensitive to acquisition modes and reconstruction parameters is recommended. The correlation of conventional metrics (SUV, MTV, etc.) and texture features should be assessed to evaluate the potential complementary value of the measures. In addition, independent validation datasets are needed to confirm the results. Finally, there is a need for easy-to-use software tools for feature extraction (their main characteristics have been very recently summarized by Hatt *et al*.^85^) since those available are handled only by non-clinician experts, are time consuming, and are able to produce a lot of textures from different matrices, many of which are probably unnecessary since their biologic significance is encapsulated within others.

**Table 4:** Summary of relevant methodological issues in calculating and reporting FDG-PET/CT texture analysis.

**All the following technical aspects should be provided for PET texture features calculation**	
a)	scanner
b)	method of images acquisition (e.g. respiratory motion, dynamic)
c)	parameters used to acquire images
d)	parameters used to reconstruct images
e)	type of images used to extract features (i.e., PET or both PET and CT)
f)	“target” of texture analysis (e.g., primary tumor, lymph nodes, metastases)
g)	application of PVC and/or a minimum lesion size/volume
h)	method of segmentation (e.g., threshold uptake 40% of the SUV_max_)
i)	discretization method (e.g., fixed number of bins)
j)	software
k)	features and matrix computation method*
**An appropriate statistical analysis should be used**	
Datasets of 10–15 patients per feature have been recommended to test the prognostic power of texture features	
**Textural features selection and validation**	
The use of the radiomics features insensitive to acquisition modes and reconstruction parameters is recommended. A correlation of conventional metrics (SUV, MTV, etc.) and texture features should be assessed to evaluate the potential complementary value of the measures. Independent validation datasets are needed to confirm the results.	

*A proposal for a consistent terminology is reported within the Supplementary material.

In conclusion, standardization is mandatory to prove the value of the information that can be derived from medical images, enabling non-invasive *in vivo* characterization of lung lesions and accurate risk stratification for the purpose of decision making regarding treatment strategy.

### Ethical approval

This article does not contain any study with human participants or animals. The patient imaged in the Fig. 3, signed an inform consent to use his personal data including imaging, also for publication; however the figure is completely anonymized, preventing the possibility of discovering the identity of the individual.

## Electronic supplementary material

Supplementary material
