# Supplementary material for: PET Radiomics in NSCLC: state of the art and a proposal for harmonization of methodology
Source: Sci Rep. 2017 Mar 23;7:358. doi: 10.1038/s41598-017-00426-y (PMC5428425; doi:10.1038/s41598-017-00426-y)

PET Radiomics in NSCLC: state of the art and a proposal for harmonization of methodology

Sollini M[1], Cozzi L[2,1], Antunovic L[3], Chiti A[1,3], Kirienko M[1]

1 Dep. Of Biomedical Sciences, Humanitas University, via Manzoni 113 - 20089 Rozzano (Milan), Italy

2 Radiotherapy and Radiosurgery Unit, Humanitas Clinical and Research Center, via Manzoni 56 - 20089 Rozzano (Milan), Italy

3 Nuclear Medicine Unit, Humanitas Clinical and Research Center, via Manzoni 56 - 20089 Rozzano (Milan), Italy

Supplementary material

Definitions of imaging features

Statistical-based

Shape and size [1,2]

To describe the shape and size of the volume of interest (V = the volume and A, the surface area of the volume of interest.)

Asphericity: is a quantitative measure of shape irregularity caused by necrotic tumor parts or invasive growth.

$$ASP = \sqrt[3]{H} - 1 \quad where\ H = \frac{1}{36\pi}\frac{A^3}{V^2}$$

Compactness 1: $\frac{V}{\sqrt{\pi}A^{2/3}}$

Compactness 2: $36\pi\frac{V^2}{A^3}$

Convex hull volume: volume of the smallest convex shape that could encompass all disease (Fried 2016)

Convexity: Measure of the spiculation of the ROI (ratio of true ROI volume to convex ROI volume)

Maximum 3D diameter: the maximum three-dimensional tumor diameter

Solidity: the volume divided by the convex hull volume

Spherical disproportion: $\frac{A}{4\pi R^2}$

(R is the radius of a sphere with the same volume as the tumor.)

Sphericity: Measure of the spherical shape (roundness) of the ROI

$$Sphericity = \frac{\pi^{1/3}(6V)^{2/3}}{A}$$

Surface to volume ratio: the surface area divided by the volume.

Total lesion glycolysis: (TLG) is given by the product of Volume and SUV_{mean}

Volume (= Metabolic Tumor Volume = Metabolic Active Tumor Volume = Metabolic Volume = Metabolic Rate Volume = Biological Target Volume = Gross Tumor Volume defined on PET images - GTV_{PET}) the sum of the volumes of all the voxels in the ROI

First-order statistics [1,3–5]

Histogram

Assume F_i to be the number of voxels with intensity I, n the total number of voxels in the volume of interest, and F the average of gray levels in the histogram.

Assume p_i to be the probability of occurrence of voxels with intensity i.

Coefficient of variation (COV): measure of the extent of variability in relation to the mean of the SUVs; it is the ratio between the standard deviation of the SUVs and the mean SUV

$Energy_{Hist}$: the sum of all voxel SUV values p squared: $\sum_i p_i^2$

$Entropy_{Hist}$: measure of uncertainty of probability distribution $-\sum_i p_i \log(p_i)$

Sample Kurtosis: measure of whether the data are heavy-tailed or light-tailed relative to a normal distribution

$$\left(\frac{1}{n}\sum_i \left(F_i - \overline{F}\right)^4\right) / \left(\frac{1}{n}\sum_i \left(F_i - \overline{F}\right)^2\right)^2$$

Mean absolute deviation: the mean of the absolute deviations of all voxel intensities around the mean intensity value

$$\frac{1}{n}\sum_i | F_i - \overline{F} |$$

Median: the median intensity value

Minimum: the minimum intensity value

Range: the range of intensity values

Root mean square: the quadratic mean, or the square root of the mean of squares of all voxel intensities.

$$F_{rms} = \sqrt{\frac{1}{n}\sum_{i} F_i^2}$$

Skewness: measure of the asymmetry of the probability distribution of intensity values about its mean

$$\left(\frac{1}{n}\sum_{i}\left(F_i - \overline{F}\right)^3\right) \Big/ \left(\frac{1}{n}\sum_{i}\left(F_i - \overline{F}\right)^2\right)^{3/2}$$

Standard deviation: the standard deviation of all SUV values $\sqrt{\frac{1}{n-1}\sum_{i}(F_i - \overline{F})^2}$

SUV_{max}: the maximum intensity value

SUV_{mean}: the mean intensity value

SUV_{peak}: the mean intensity value within a 1 cm^3 sphere centered around the maximum SUV voxel

Uniformity: $\sum_{i} F_i^2$

$Variance_{Hist}$: indicates how widely the SUVs vary: $\frac{1}{n}\sum_{i}\left(F_i - \overline{F}\right)^2$

I_x: minimum intensity to x% highest intensity volume

V_x: percentage volume having at least x% intensity value

Area under the IVH (or CSH) (AUC-IVH or AUC-CSH): is the area under the curve (definite integral) of the plot of the percent volume of a tumor with a SUV above a certain threshold, which is varied from 0% to 100% of SUV_{max}

Second-level statistics [3,5–8]

The gray-level co-occurrence matrix features (GLCM) (gray level = gray tone)

The i,j^{th} element of the co-occurrence matrix for an anatomical structure of interest represents the number of times that intensity levels i and j occur in two voxels separated by distance (d) in direction (a). The co-occurrence features are based on the second-order joint conditional probability density function $P(i,j;\, a,d)$ of a given texture image.

These metrics are independent of tumor position, orientation, size, and intensity and take into account the spatial distribution of the local voxel intensities.

Assumption: $p(i,j)$ is the joint probability of co-occurring pixel intensity values i and j. N_x is the number of pixel intensities.

$$p_{x+y(}(k) = \sum_i \sum_j p(i,j), \quad i+j=k \qquad p_{x-y(}(k) = \sum_i \sum_j p(i,j), \quad |i-j| = k$$

$$Angular\ Second\ Moment \quad AngScMom = \sum_i \sum_j p(i,j)^2$$

$$Entropy_{GLCM} = -\sum_i \sum_j p(i,j)\log(p(i,j)$$

$$Inverse Difference Moment \quad InvDfMom = \sum_i \sum_j \frac{1}{1+(i-j)^2} p(i,j)$$

$$SumOf\ Squares \quad SumOfSqs = \sum_i \sum_j (i-\mu_x)^2 p(i,j)$$

$$SumEntropy \quad SumEntrp = -\sum_i^x p_{x+y}(i)\log(p_{x+y}(i,j)$$

$$SumAverage \quad SumAverg = \sum_i i p_{x+y}(i)$$

$$SumVariance \quad SumVarnc = \sum_i (i - sumAverg)^2 p_{x+y}(i)$$

$$Differenceentropy \quad DifEntrp = -\sum_i p_{x-y}(i)\log(p_{x-y}(i,j)$$

$$DifferenceVariance \quad DifVarnc = \sum_i (i-\mu_{x-y})^2 p_{x-y}(i,j)$$

$$Autocorrelation = \sum_i \sum_j ijp(i,j)$$

$$Cluster\ pro\min ence = \sum_i \sum_j ((i+j-\mu_x(i))^4 p(i,j)$$

$$ClusterShade_{GLCM} = \sum_i \sum_j (i+j-\mu_x(i)-\mu_y(j))^3 p(i,j)$$

$$Cluster\ Tendency = \sum_i \sum_j (i+j-\mu_x(i)-\mu_y(j))^2 p(i,j)$$

$$Contrast_{GLCM} = \sum_i \sum_j (i-j)^2 p(i,j)$$

$$Correlation_{GLCM} = \sum_{i=1}^{N_x} \sum_{j=1}^{N_x} (i-\mu_x(i))(j-\mu_x(j))p(i,j)/(\sigma_x \sigma_y)$$

Diagonal moment: measures the differences in correlation for high and low gray levels

$$D = \sum_i \sum_j |i-j|(i+j-\mu_x-\mu_y)C(i,j)$$

Dissimilarity: $\sum_i \sum_j |i-j|\, C(i,j)$

Energy$_{\text{GLCM}}$: $\sum_i \sum_j C(i,j)^2$

Homogeneity 1: $\sum_i \sum_j C(i,j)/(1+|i-j|)$

Homogeneity 2: $\sum_i \sum_j p(i,j)/(1+|i-j|^2)$

Informational measure of correlation 1: (H is Entropy)

$$IMC1 = \frac{H - HXY1}{\max\{HX, HY\}}$$

Informational measure of correlation 2: (H is Entropy)

$$IMC2 = \sqrt{1 - e^{-2(HXY2 - H)}}$$

Inverse difference moment normalized:

$$IDMN = \sum_i \sum_j \frac{p(i,j)}{1 + \frac{|i-j|^2}{N^2}}$$

Inverse difference normalized:

$$IDN = \sum_i \sum_j \frac{p(i,j)}{1 + \frac{|i-j|}{N^2}}$$

Maximum probability:

Maximum probability = $\max\{p(i,j)\}$

Second diagonal moment: Digital image processing in machining.

$$SDM = \sum_i \sum_j 0.5\,|i-j|\, p(i,j)$$

Variance$_{GLCM}$: $\sum_i \sum_j (i-\mu)\, p(i, j)$

High-order features [3,5–8]

Gray-level run-length matrix-based features (GLRLM)

Let $p(i, j|q)$ be the (i, j)th entry in the given run-length matrix p for a direction q,

Ng the number of discrete intensity values in the image, Nr the number of different run lengths, and Np the number of voxels in the image.

Short-Run Emphasis (SRE): $SRE = \dfrac{\sum_i \sum_j \left[\dfrac{p(i, j \mid \theta)}{j^2} \right]}{\sum_i \sum_j p(i, j \mid \theta)}$

Long-Run Emphasis (LRE): $LRE = \dfrac{\sum_i \sum_j j^2 p(i, j \mid \theta)}{\sum_i \sum_j p(i, j \mid \theta)}$

Gray-Level Non-Uniformity (for run)(GLN(r)): $GLN = \dfrac{\sum_i \left[\sum_j p(i, j \mid \theta) \right]^2}{\sum_i \sum_j p(i, j \mid \theta)}$

Run Length Non-Uniformity (RLN): $RLN = \dfrac{\sum_{j=1} \left[\sum_{i=1} p(i, j \mid \theta) \right]^2}{\sum_i \sum_j p(i, j \mid \theta)}$

Run Percentage (RP): $RP = \dfrac{\sum_i \sum_j p(i, j \mid \theta)}{N}$

Low Gray-Level Run Emphasis (LGLRE): $LGLRE = \dfrac{\sum_i \sum_j \left[\dfrac{p(i, j \mid \theta)}{j^2} \right]}{\sum_i \sum_j p(i, j \mid \theta)}$

High Gray-Level Run Emphasis (HGLRE): $HGLRE = \dfrac{\sum_i \sum_j i^2 p(i, j \mid \theta)}{\sum_i \sum_j p(i, j \mid \theta)}$

Short-Run Low Gray-Level Emphasis (SRLGLE): $SRLGLE = \dfrac{\sum_i \sum_j \left[\dfrac{p(i, j \mid \theta)}{i^2 j^2} \right]}{\sum_i \sum_j p(i, j \mid \theta)}$

Short-Run High Gray-Level Emphasis (SRHGLE): $SRHGLE = \dfrac{\sum_i \sum_j \left[\dfrac{i^2 p(i, j \mid \theta)}{j^2} \right]}{\sum_i \sum_j p(i, j \mid \theta)}$

Long-Run Low Gray-Level Emphasis (LRLGLE): $LRLGLE = \dfrac{\sum_i \sum_j^{x} \left[\dfrac{j^2 p(i, j \mid \theta)}{i^2} \right]}{\sum_i \sum_j p(i, j \mid \theta)}$

Long-Run High Gray-Level Emphasis (LRHGLE): $LRHGLE = \dfrac{\sum_i \sum_j i^2 j^2 p(i, j \mid \theta)}{\sum_i \sum_j p(i, j \mid \theta)}$

Gray-level size-zone matrix-based features (GLSZM)

Let:

$p(i, j)$ be the $(i, j)^{th}$ entry in the given size-zone matrix p,

Ng the number of discrete intensity values in the image,

Nz the size of the largest, homogeneous region in the volume of interest,

Na the number homogeneous areas in the image.

Small-area emphasis (SAE) or short-zone emphasis (SZE): $SAE = \frac{\sum_i \sum_j \left[\frac{p(i,j)}{j^2} \right]}{\sum_i \sum_j p(i,j)}$

Large-area emphasis (LAE) or long-zone emphasis (LZE): $LAE = \frac{\sum_i \sum_j j^2 p(i,j)}{\sum_i \sum_j p(i,j)}$

Intensity variability (IV): $IV = \frac{\sum_i \left[\sum_j p(i,j) \right]^2}{\sum_i \sum_j p(i,j)}$

Gray-level non-uniformity for zone (GLUNz): $GLNUz = \frac{1}{\theta} \sum_i \left[\sum_j Z(i,j) \right]^2$

Zone length non-uniformity (ZLN): $GLNUz = \frac{1}{\theta} \sum_j \left[\sum_i Z(i,j) \right]^2$

Size-zone variability (SZV): $SVZ = \frac{\sum_j \left[\sum_i p(i,j) \right]^2}{\sum_i \sum_j p(i,j)}$

Zone percentage (ZP): $ZP = \frac{\sum_i \sum_j p(i,j)}{N}$

Weighted variance of gray-level size, *N* direction (WVGLS_N):

$$Var_N = \sqrt{\frac{1}{NS} \sum_n \sum_s \left(nM(n,s) - \mu_N \right)^2} \quad with\ \mu_N = \frac{1}{NS} \sum_n \sum_s nM(n,s)$$

Weighted variance of gray-level size, *S* direction (WVGLS_S):

$$Var_S = \sqrt{\frac{1}{NS} \sum_n \sum_s \left(sM(n,s) - \mu_S \right)^2} \quad with\ \mu_S = \frac{1}{NS} \sum_n \sum_s sM(n,s)$$

Low-intensity emphasis (LIE) or low gray-level zone emphasis (LGZE):

$$LIE = \frac{\sum_{i=1}\sum_{j=1}\left[\frac{Z(i,j)}{i^2}\right]}{\sum_i\sum_j Z(i,j)}$$

High-intensity emphasis (HIE) or high gray-level zone emphasis (HGZE):

$$HIE = \frac{\sum_i\sum_j i^2 Z(i,j)}{\sum_i\sum_j Z(i,j)}$$

Low-intensity small-area emphasis (LISAE) or short-zone low gray-level emphasis (SZLGE):

$$LISAE = \frac{\sum_i\sum_j \frac{Z(i,j)}{i^2 j^2}}{\sum_i\sum_j Z(i,j)}$$

High-intensity small-area emphasis (HISAE) or short-zone high gray-level emphasis (SZHGE):

$$HISAE = \frac{\sum_i\sum_j \frac{i^2 Z(i,j)}{j^2}}{\sum_i\sum_j Z(i,j)}$$

Low-intensity large-area emphasis (LILAE) or long-zone low gray-level emphasis (LZLGE):

$$LILAE = \frac{\sum_i\sum_j \frac{j^2 Z(i,j)}{i^2}}{\sum_i\sum_j Z(i,j)}$$

High-intensity large-area emphasis (HILAE) or long-zone high gray-level emphasis (LZHGE):

$$HILAE = \frac{\sum_i\sum_j \frac{i^2 j^2 Z(i,j)}{i^2}}{\sum_i\sum_j Z(i,j)}$$

Neighborhood gray-level (or gray-tone) different matrix (NGLDM or NGTDM) features

Coarseness: $\frac{1}{\sum_i N(i,1)N(i,2)}$

Contrast$_{NGTDM}$: $\sum_i \sum_j N(i,1)N(j,1)(i-j)^2 \cdot \sum_i N(i,2)/En(n-1)$

Busyness: $\sum_i \sum_j N(i,1)N(i,2)/\sum_i \sum_j (iN(i,1)-jN(j,1))\ with\ N(i,1)\neq 0\ N(j,1)\neq 0$

Complexity:

$$\sum_i \sum_j \{(|i-j|)/(n^2(p_i+p_j))\}\{p_i s(i)+p_j(s(j)\}$$

Texture strength (TS): $\frac{\sum_i \sum_j (p_i+p_j)(i-j)^2}{\varepsilon+\sum_i s(i)}$

Neighboring gray-level dependence matrix (NGLDM) features

Small number emphasis (SNE): is a measure of the fineness of the image

$$SNE=\sum_k \sum_s (Q(k,s))/s^2/R$$

Large number emphasis (LNE): is a measure of the coarseness of the image

$$LNE=\sum_k \sum_s s^2(Q(k,s))/R$$

Number non-uniformity (NN):

$$NNU=\sum_k \sum_s (Q(k,s))/R$$

Second moment (SM): is a measure of the homogeneity of a Q matrix and therefore of the image

$$SM = \sum_k \sum_s \left(Q(k,s)\right)^2 / R$$

Entropy$_{NGLDM}$:

$$ENT_{NGLDM} = \sum_k \sum_s \left(Q(k,s)\right) \log\left(\left(Q(k,s)\right)\right) / R$$

Absolute Gradient based features [9]

For the gradient feature calculation the following neighborhood for image pixel *x*(*i*,*j*) is defined:

$$\begin{array}{ccccc} A & B & C & D & E \\ F & G & H & I & J \\ K & L & x(i,j) & N & O \\ P & Q & R & S & T \\ U & V & W & Y & Z \end{array}$$

Based on this neighborhood, the absolute gradient value (ABSV(*i*,*j*)) is calculated for each pixel:

a) for 5x5 pixel neighborhood:

$$ABSV5(i,j) = \sqrt{(W-C)^2 + (O-K)^2}$$

b) for 3x3 pixel neighborhood:

$$ABSV3(i,j) = \sqrt{(R-H)^2 + (N-L)^2}$$

For the ABSV=ABSV3 matrix of *M* elements (which contains absolute gradient values for ROI pixels), the gradient features are defined as follows:

Absolute gradient value *G*(*i*,*j*) for 5x5 matrix of *M* elements:

$$G(i,j) = \sqrt{(x_{i+2,j} - x_{i-2,j})^2 + (x_{i,j+2} - x_{i,j-2})^2}$$

Mean of *G*(*i*,*j*); $GrMean = \frac{1}{M} \sum_{i,j} G(i,j)$

Variance of $G(i,j)$ $GrVariance = \frac{1}{M}\sum_{i,j}(G(i, j - GrMean)^2$

Ratio of non-zero $G(i,j)$ elements: GrNonZeros=Ratio of non-zero $G(i,j)$ values

Skewness of absolute gradient:

$$GrSkewness = \frac{1}{(GrVariance)^{3/2}}\frac{1}{M}\sum_{i,j}(ABSV(i, j) - GrMean)^3$$

Kurtosis of absolute gradient:

$$GrKurtosis = \frac{1}{(GrVariance)^{4}}\frac{1}{M}\sum_{i,j}(ABSV(i, j) - GrMean)^4 - 3$$

Spatial Autocorrelation [10]

This includes indices such as Moran's index and Geary's coefficient, which attempt to identify whether spatial autocorrelation exists for a single variable. The Moran's index and Geary's coefficient summarize the strength of associations between responses as a function of distance and possibly direction. This coefficient does not provide the same information as spatial autocorrelation given by Moran's index, because it emphasizes the differences in values between pairs of observations rather than the covariation between the pairs. So the Moran's index gives a more global indicator whereas the Geary's coefficient is more sensitive to differences in small neighborhoods. Moran's index is one of the oldest indicators of spatial autocorrelation. It is applied to zones or points which have continuous variables associated with their intensities. For any continuous variable, *xi*, a mean can be calculated and the deviation of any observation from that mean can also be calculated. The statistic then compares the value of the variable at any one location with the value at all other locations. It is formally defined as:

$$I(h) = \frac{N\sum_{i}\sum_{j} w_{ij}(x_i - \bar{X})(x_j - \bar{X})}{\sum_{i}\sum_{j} w_{ij}\sum_{i}(x_i - \bar{X})^2}$$

where h is the lag vector, N is the number of cases, x_i (target or head voxel value) is the variable value at a particular location i, x_j (source or tail voxel value) is the variable value at another location, X is the mean of the variable, and w_{ij} is a weight applied to the comparison between location I and location j. In more current use, w_{ij} is a distance-based weight which is the inverse distance between locations i and j.

The Geary's coefficient takes into account that the interaction is not the cross product of the deviations from the mean but the deviation in intensities of each observation location with another one. Its formal definition is:

$$C(h) = \frac{(N-1)\sum_i \sum_j w_{ij}(x_i - x_j)^2}{\sum_i \sum_j w_{ij} \sum_i (x_i - \bar{X})^2}$$

The values of *C* typically vary between 0 and 2. The theoretical value of *C* is 1, which indicates that values of one zone are spatially unrelated to the values of any other zone. Values less than 1 indicate a positive spatial autocorrelation while values greater than 1 indicate a negative spatial autocorrelation.

Model-based

Autoregression Models [9,11,12]

The linear dependence that one pixel of an image has on another is well known and can be illustrated by the autocorrelation function.

A pixel *(i,j)* depends on a two-dimensional neighborhood *N(i,j)* consisting of pixels above or to the left of it as opposed to the simple sequence of the previous pixels a raster scan could define. For each pixel *(k,I)* in an order-D neighborhood for pixel (i, *j), (k,I)* must be previous to pixel *(i,j)* in a standard raster sequence and *(k , I)* must not have any coordinates more than *D* units away from*(i,j)*. Formally, the order-D neighborhood is defined by:

$$N(i,j) = \{(k,l \mid (i - D \le k < i \; and \; j - D \le l \le j + D) \; or \; (k = i \; and \; j - D \le l < j)\}$$

Supplementary Figure 1 is an illustration of how, from a randomly generated noise image and a given starting sequence $a_1, \ldots a_k$, representing the initial boundary conditions, all values in a texture image can by synthesized by a one-dimensional autoregressive model (modified from Haralick[12]).

$$a(i,j) = \sum_{k,l} \alpha(i-k, l-j) a(k,l) + \sum_{k,l} \beta(i-k, l-j) b(k,l)$$

where the first is the autoregressive term and the second the moving average term.

The autoregressive (AR) model assumes a local interaction between image pixels in that pixel intensity is a weighted sum of neighboring pixel intensities. Assuming image f is a zero-mean random field, an AR causal model can be defined as:

$$f_s = \sum_r \theta_r f_r + e_s$$

where f_s is image intensity at site *s*, e_s denotes an independent and identically distributed (i.i.d.) noise, N_s is a neighborhood of *s*, and θ is a vector of model parameters. Using the AR model for image segmentation consists in identifying the model parameters for a given image region and then using the obtained parameter values for texture discrimination. In the case of a simple pixel neighborhood, that comprises four immediate pixel neighbors, there are five unknown model parameters – the standard deviation σ of the driving noise e_s and the model parameter vector θ= [θ1,θ2,θ3,θ4]. The parameters can be estimated by minimizing the sum of the squared error

$$\sum_s e_s^2 = \sum_s (f_s - w_s)^2$$

which leads to the following linear equations:

$$\hat{\theta} = \left(\sum_s w_s w_s^T \right)^{-1} \left(\sum_s w_s f_s \right) \quad \sigma^2 = N^{-2} \sum_s (f_s - w_s)^2$$

where w_s = col[fi, $i \in N_s$], and the square $N \times N$ image is assumed.

Fractal features [13]

Fractal dimension: is a representation of how an object fills space.

Fractal geometry is characterized by the relationship between a measure (M) and a scale (ε), expressed as:

$M(\varepsilon) = k \cdot \varepsilon^{-D}$,

where k is a scaling constant and D is the fractal dimension that is used to detect self-affinity. The morphological fractal dimension (m-FD) is a quantitative index of morphological complexity derived from CT on PET/CT, with higher values corresponding to increasing degrees of complexity. The heterogeneity of FDG distribution is expressed as the density fractal dimension (d-FD), of which higher values correspond to increasing degrees of heterogeneity. In d-FD, the chosen cut-offs were used as the ruler scale ε in the above equation. The number of voxels containing radioactivity higher than the corresponding cut-offs is expressed as $M(\varepsilon)$, in which M decreases as ε increases and the magnitude of the slope of linear regression between the logarithms of the cut-offs and the number of pixels is equal to the fractal dimension.

Fractal dimension and fractal abundance were calculated using a box-counting method, with multiple grid offsets for all possible box start locations, based on the following equation:

$N_L = KL^{-D}$

where L is the box size, N_L is the number of boxes of size L needed to cover the object being studied, and D is the fractal dimension. By plotting a log-log plot of N_L versus L, fractal dimension (FD) can be obtained from the slope, and fractal abundance (FA) or log K can be obtained from the y-intercept of the straight portion of the curve.

Abundance: represents the volume of space explored

Lacunarity: a measure of structural heterogeneity within an object

Lacunarity was derived using a gliding box method defined by the equation:

$$\Lambda = \sum s^2 Q(s,r) \Big/ \left(\sum sQ(s,r)\right)^2$$

where Λ represents lacunarity, r represents box size, s represents the number of occupied sites within a box size, and Q is the probability distribution (representing the frequency distribution of the total number of occupied sites for a box size r over the total number of boxes of size r). Lacunarity is displayed by plotting a log-log plot of lacunarity versus size of the gliding box. Lacunarity thus provides a dimensionless representation of the fraction of sites that is occupied.

Transform-based

Wavelet transform [14,15]

Wavelet transform is an improved tool of frequency transform for multi-resolution processing. A bank of filters are applied on images to obtain the image information at different resolution levels.. Wavelets compute average intensity properties as well as several detailed contrast levels distributed throughout the image. The discrete wavelet transform iteratively decomposes an image into four components based on frequency content and orientation. Wavelet transform can decompose an image into four lower resolution images containing low-frequency components in both directions (LL), high-frequency component in x-direction and low-frequency component in y-direction (HL), low-frequency component in x-direction and high-frequency component in y-direction (LH), and high-frequency component in both directions (HH).

The general mother wavelet can be constructed from the following scaling $\phi(x)$ and wavelet functions $\psi(x)$:

$$\phi(x) = \sqrt{2}\sum h(k)\, \phi(2x - k)$$

$$\psi(x) = \sqrt{2}\sum g(k)\, \phi(2x - k)$$

where $g(k) = -1kh(N - 1 - k)$, and N is the number of scaling and wavelet coefficients. The sets of scaling ($h(k)$) and wavelet ($g(k)$) function coefficients vary depending on their corresponding wavelet bases.

Laws family [16]

Laws features are constructed from a set of five one-dimensional filters, each designed to respond to a different type of structure in the image. These one-dimensional filters are denoted E5 (edges), S5 (spots), R5 (ripples), W5 (waves), and L5 (low-pass, or average gray value). By applying a one-dimensional filter in the horizontal direction followed by a (possibly different) one-dimensional filter in the vertical direction, results from 25 different two-dimensional filters can be calculated. These filters were applied to the image at full resolution and to the image downsized by factors of 2, 4, 8, and 16 using cubic spline interpolation. The downsized images allow the Laws filters to detect increasingly broader and coarser textures. The second and fourth moments within the CTR for each

of the 25 filters were then calculated. Thus a total of 25*5*2 = 250 Laws features were calculated for each image.

References of Supplementary Material

1. Leijenaar, R. T. H. *et al.* Stability of FDG-PET Radiomics features: an integrated analysis of test-retest and inter-observer variability. *Acta Oncol.* **52,** 1391–7 (2013).

2. Apostolova, I. *et al.* Quantitative assessment of the asphericity of pretherapeutic FDG uptake as an independent predictor of outcome in NSCLC. *BMC Cancer* **14,** 896 (2014).

3. Yan, J. *et al.* Impact of Image Reconstruction Settings on Texture Features in 18 F-FDG PET. *J nucl med* **56,** 1667–1674 (2015).

4. El Naqa, I. *et al.* Exploring feature-based approaches in PET images for predicting cancer treatment outcomes. *Pattern Recognit.* **42,** 1162–1171 (2009).

5. Cheng, N. *et al.* Textural features of pretreatment 18F-FDG PET / CT images : prognostic significance in patients with advanced T-stage oropharyngeal squamous cell carcinoma . PubMed Commons. *J Nucl Med* **54,** 1709–9 (2013).

6. Orlhac, F. *et al.* Tumor Texture Analysis in 18F-FDG PET: Relationships Between Texture Parameters, Histogram Indices, Standardized Uptake Values, Metabolic Volumes, and Total Lesion Glycolysis. *J. Nucl. Med.* **55,** 414–422 (2014).

7. Tixier, F. *et al.* Visual Versus Quantitative Assessment of Intratumor 18F-FDG PET Uptake Heterogeneity: Prognostic Value in Non-Small Cell Lung Cancer. *J. Nucl. Med.* **55,** 1235–1241 (2014).

8. Dutta, S., Pal, K. & Sen, R. in *Mod. Mech. Eng. Res. Dev. Educ.* (ed. Davim, P.) 367–410 (Springer-Verlag Berlin Heidelberg, 2014). doi:10.1007/978-3-642-45176-8

9. Ha, S. *et al.* Autoclustering of Non-small Cell Lung Carcinoma Subtypes on 18F-FDG PET Using Texture Analysis: A Preliminary Result. *Nucl. Med. Mol. Imaging (2010).* **48,** 278–286 (2014).

10. Da Silva, E. C., Silva, A. C., De Paiva, A. C. & Nunes, R. A. Diagnosis of lung nodule using Moran’s index and Geary's coefficient in computerized tomography images. *Pattern Anal. Appl.* **11,** 89–99 (2008).

11. Haralick, R., Shanmugam, K. & Dinstein, I. Texture Features for Image Classification. *IEEE Trans Sys Man Cyb SMC.* **3,** 610–621 (1973).

12. Haralick, R. M. Statistical and Structural Approaches To Texture. *Proc IEEE* **67,** 786–804 (1979).

13. Goh, V., Sanghera, B., Wellsted, D. M., Sundin, J. & Halligan, S. Assessment of the spatial pattern of colorectal tumour perfusion estimated at perfusion CT using two-dimensional fractal analysis. *Eur. Radiol.* **19,** 1358–1365 (2009).

14. Dettori, L. & Semler, L. A comparison of wavelet, ridgelet, and curvelet-based texture classification algorithms in computed tomography. *Comput. Biol. Med.* **37,** 486–498 (2007).

15. Wu, J. & Rubin, D. L. Early-Stage Non – Small Cell Lung Cancer : Quantitative Imaging Characteristics of 18 F Fluorodeoxyglucose PET / CT Allow. *Radiology* (2016).

16. Manduca, A. *et al.* Texture features from mammographic images and risk of breast cancer. *Cancer Epidemiol. Biomarkers Prev.* **18,** 837–845 (2009).

Supplementary legend for figure

Supplementary Figure 1: illustration of how, from a randomly generated noise image and a given starting sequence $a_1,\ldots a_k$, representing the initial boundary conditions, all values in a texture image can by synthesized by a one-dimensional autoregressive model (modified from Haralick[12]).

Supplementary figure 1

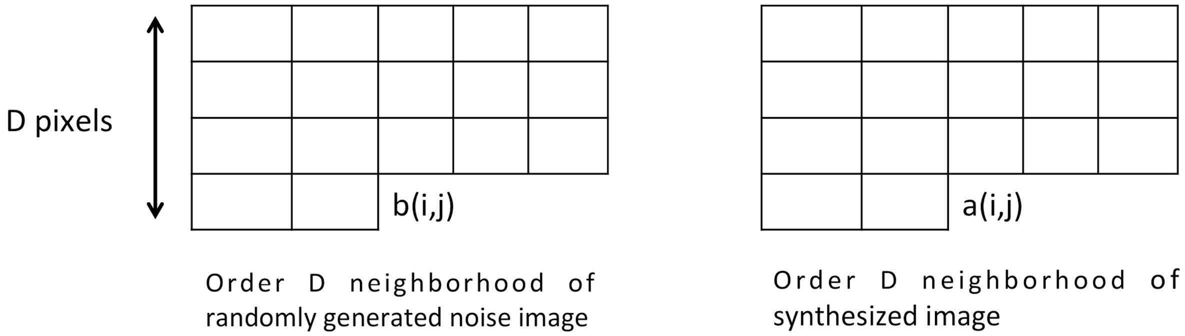

Supplement: Supplementary file 1 — Supplementary material [file 41598_2017_426_MOESM1_ESM.pdf]
